# Insights into the association of *Nicotiana tabacum* health with eukaryotic microbial community and environmental factors

**DOI:** 10.3389/fpls.2025.1563283

**Published:** 2025-04-03

**Authors:** Longxin Chai, Yue Shun, Lei Xue, Yong Yang, Mei Li

**Affiliations:** State Key Laboratory of Biocatalysis and Enzyme Engineering, School of Life Sciences, Hubei University, Wuhan, Hubei, China

**Keywords:** *Nicotiana tabacum*, eukaryotic microbial community, health status, soil condition, machine learning, environmental factor

## Abstract

As an important cash crop, *Nicotiana tabacum’s* yield and quality are influenced by various factors, yet the correlations between its health status, microbial community, and environmental factors remain largely unexplored. In this study, we analyzed the microbial diversity of *Nicotiana tabacum* rhizosphere microbiomes using ITS rDNA sequencing under different conditions. Compared with soil associated with healthy *Nicotiana tabacum*, the alpha and beta diversity of the eukaryotic microbial community decreased in soil with diseased *Nicotiana tabacum*, indicating a decline in microbial abundance and composition. Compared with healthy soil, the eukaryotic microbial community in diseased soil exhibited looser structural networks, with the assembly process of both communities predominantly governed by stochastic processes. Soil element measurements and correlation analyses identified pH, manganese, and copper as key environmental factors associated with the health status of *Nicotiana tabacum.* A machine learning model incorporating environmental factors and major microbial phyla was developed to predict *Nicotiana tabacum* health status, achieving a high accuracy of 93%. These findings collectively offer comprehensive insights into the relationship between *Nicotiana tabacum* health status, soil conditions, environmental factors, and eukaryotic microbial community.

## 1 Introduction

The plant-associated microbial community, characterized by its complex composition and vast numbers, plays a vital role in the health of land plants and is often regarded as their “second genome” (Tringe et al., 2005; Berendsen et al., 2012). Symbiotic microbial communities facilitate nutrient exchange with land plants, promoting mutual growth (Long, 1989; Bolan, 1991; Zhang et al., 2009). Additionally, rhizosphere microbes contribute to pathogen resistance, water retention, and the synthesis of growth-promoting hormones (Mendes et al., 2011; Bulgarelli et al., 2013). Root-associated microbial communities, present in both the rhizosphere and endosphere compartments, are strongly influenced by plant development, primarily through the effects of root exudates (Trivedi et al., 2020).

The plant-associated microbial community exhibits high diversity and undergoes change based on plant health status (Song et al., 2020; Adedayo et al., 2022). In addition to bacterial microbes, land plants host numerous soil-borne and filamentous eukaryotic microbes, including fungi and oomycetes (Ruggiero et al., 2015; Duran et al., 2018; Sutela et al., 2019). Notably, fungi interact with plants in various ways, with each interaction leading to distinct alterations in both partners (Zeilinger et al., 2016; Tripathi et al., 2022; Mishra et al., 2024). Oomycota constitutes a distinct class of fungus-like eukaryotic microbes, with many species acting as highly destructive plant or animal pathogens (Fawke et al., 2015).

Complete and balanced nutrition is crucial for both plant growth and defense against invading pathogens (Tripathi et al., 2022). Mineral nutrients—including primary macronutrients, secondary macronutrients, and micronutrients—influence plant health by regulating enzyme activity and indirectly enhancing plant vigor through various pathways (Tripathi et al., 2022). Primary macronutrients such as nitrogen (N), phosphorus (P), and potassium (K) collectively regulate plant defense mechanisms. While N contributes to phytoalexin biosynthesis and K mediates stomatal immunity (Bolton and Thomma, 2008; Tripathi et al., 2022; Ortel et al., 2024), P plays a crucial role in energy metabolism and signal transduction during pathogen invasion (Zipfel and Oldroyd, 2017; Chan et al., 2021). The micronutrient manganese is essential for photosynthesis, lignin biosynthesis, and other plant metabolic functions (Graham, 1983). Zinc is involved in auxin synthesis, as well as the production of infectivity factors, phytotoxins, and mycotoxins in pathogenic microorganisms (Dordas, 2008). Copper is an essential trace element involved in various cellular processes, including protein transport, cell wall metabolism, and photosynthesis, making it crucial for the regular growth and development of plants (Chen G. et al., 2022). More importantly, these elements can either enhance or reduce host susceptibility to disease onset and progression (Tripathi et al., 2022). In addition, soil pH has been reported to significantly influence soil biological, chemical, and physical processes in the natural environment (Neina, 2019).

*Nicotiana tabacum*, a land plant and cash crop that thrives in diverse growing environments, is susceptible to various diseases at different growth stages (Yuan et al., 2018). Common diseases of *Nicotiana tabacum* include viral, bacterial, fungal, and soil-borne diseases, which can significantly reduce its yield and quality (Ahmed et al., 2022). Recent studies using high-throughput sequencing have provided new insights into the bacterial composition and organization of different plant microbiomes, including *Arabidopsis*, *Populus*, and maize (Gottel et al., 2011; Bulgarelli et al., 2012; Lundberg et al., 2012; Peiffer et al., 2013; Shakya et al., 2013; Schlaeppi et al., 2014). Despite advances in microbial research, little is known regarding how the totality of *Nicotiana tabacum* rhizosphere microbes, particularly the eukaryotic microbial community, is shaped by various factors or the plant’s health status. To explore how the microbial community and environmental factors correlate with the health status of *Nicotiana tabacum*, we conducted microbial diversity analyses of its rhizosphere microbiome to identify key environmental factors and phyla. Additionally, a machine learning model was built to predict health status.

## 2 Materials and methods

### 2.1 Collection and processing of *Nicotiana tabacum* rhizosphere soil samples

The study area is in Xiaogan region (30°54′–31°46′N, 113°19′–114°35′E), Hubei Province, China, characterized by a humid subtropical monsoon climate with an annual average temperature of 16.2°C and precipitation of 1,100 mm. The sampling sites, located at elevations of 500, 900, and 1,300 m, are in hilly regions with predominantly yellow-brown soils (pH 5.8–6.5) used for continuous tobacco cultivation. At each elevation, three plots (10 m × 10 m) representing different health statuses were selected: healthy (no visible disease symptoms in plants), diseased (typical soil-borne disease symptoms in plants), and control (fallow fields without tobacco cultivation for ≥ 3 years). Each health condition at each elevation was represented by three replicate plots (10 m × 10 m), resulting in 27 sampling points (3 elevations × 3 health statuses × 3 replicates). Within each plot, five 1 m × 1 m subplots were arranged diagonally, and three subplots were randomly selected for sampling, yielding a total of 81 samples (27 points × 3 subplots). Rhizosphere soil (0–20 cm depth) was collected by gently shaking the roots to remove loosely adhered soil, followed by brushing with sterile spatulas to obtain tightly bound rhizosphere soil (Peiffer et al., 2013). After collection, the samples were homogenized in sterile phosphate-buffered saline (PBS, pH 7.4) at a 1:5 (w/v) ratio by vortex mixing (2,000 rpm, 10 min), followed by filtration through a 2-mm filter to remove root debris. The homogenized suspensions were aliquoted for DNA extraction using the Cetyltrimethylammonium Bromide (CTAB) method and stored at − 80°C (Bulgarelli et al., 2012; Rastogi et al., 2012).

### 2.2 Measurement of environmental factors

To investigate the potential drivers of microbial composition changes, we conducted detailed measurements of soil physical and chemical properties. Soil pH was measured using a LeiCi PHSJ-4F pH meter (INESA, Shanghai, China) with a 1:2.5 soil-to-water ratio after 30 min of equilibration. Alkaline nitrogen (AN) was determined by the diffusion absorption method, while organic matter (OM) was quantified using the potassium dichromate external heating method. Soil samples were digested with 0.8 mol/L K_2_Cr_2_O_7_ and concentrated H_2_SO_4_ at 180°C for 5 min, then titrated with FeSO_4_. Total nitrogen (TN) was analyzed using a FlashSmart Elemental Analyzer (Italy) via the Dumas combustion method. The soil was combusted at 900°C under a He flow of 200 mL/min, and N_2_ was quantified by thermal conductivity detection. Available phosphorus (AP) was extracted with 0.5 mol/L NaHCO_3_ (pH 8.5) and determined by molybdenum-blue colorimetry using UV-1800PC UV-Vis Spectrophotometer (MAPADA, Shanghai, China) at 700 nm. Available potassium (AK) was extracted with 1 mol/L NH_4_OAc (pH 7.0) and measured by flame atomic absorption spectrometry (AAS FP6410, INESA, Shanghai, China) at 766.5 nm. Exchangeable Ca/Mg and available Fe/Mn/Cu/Zn were extracted with 0.005 mol/L DTPA (pH 7.3) and analyzed by inductively coupled plasma mass spectrometry (ICP-MS 7900, Agilent, California, USA). Quality control included triplicate measurements, the use of certified reference materials (GSS-8 for soil), and blank corrections. All data are provided in **Supplementary Table S1**.

### 2.3 DNA extraction and ITS rDNA sequencing and analysis

DNA from soil samples was extracted using either the CTAB or SDS method, validated via agarose gel electrophoresis, and diluted into 1 ng/µL. Using the diluted genomic DNA as a template, specific primers with barcode tags, ITS5-1737F (5′-GGAAGTAAAAGTCGTAACAAGG-3′) and ITS2-2043R (5′-GCTGCGTTCTTCATCGATGC-3′), were used to amplify the internal transcribed spacer (ITS) ribosomal DNA (rDNA) gene. The PCR product was subjected to 2% agarose gel electrophoresis and gel purification. Subsequently, the TruSeq DNA PCR-Free Sample Preparation Kit was used for library preparation. The constructed library was quantified using Qubit and qPCR and subjected to high-throughput sequencing with HiSeq2500 PE250.

For the reads of each sample, FLASH (Magoc and Salzberg, 2011) was used for splicing, and Qiime V1.9.1 (Caporaso et al., 2010) was used for quality control of the spliced sequences and removal of chimera sequences to obtain effective tags (Edgar, 2018). Uparse software v7.0.1001 (Edgar, 2013) was then used to cluster the valid tags from all samples. By default, sequences were clustered into operational taxonomic units (OTUs) with 97% identity. Representative OUT sequences were selected, and species annotation was performed using the BLAST method in Qiime V 1.9.1 and the UNITE database (Koljalg et al., 2020).

### 2.4 Microbial metagenomics analysis

Microbial metagenomics analysis was conducted using various R packages. Specifically, alpha diversity, indicated by the Shannon diversity index, was calculated using *estimateR* and *diversity* functions from the *vegan* package. Beta diversity, based on Bray–Curtis dissimilarity, was assessed using the *anosim* function from the *vegan* package. Statistical significance for alpha and beta diversity among samples was determined using the *ducan.test* function from the *agricolae* package.

To estimate the community structure and relationships of eukaryotic microbes under different soil conditions, OTUs with relative abundance values ≥ 0.02% in each soil condition were selected to construct the co-occurrence network of the eukaryotic microbial community. The *igraph* package was used to calculate the topological characteristics of the subnetwork in each sample, including the average degree (the average number of connections per node, reflecting network complexity), average path length (the mean shortest path between nodes, measuring network compactness), betweenness centrality (an indicator of a node’s ability to control information flow within the network), and closeness centrality (an indicator of the proximity of a node’s connections within the network) among fungal microorganisms. The *WGCNA* package was used to organize and integrate correlation and significance information between OTUs, providing foundational data for subsequent network construction or analysis. The *ggplot2* package was used to visualize the network.

Null model analysis (Gotelli, 2000) was performed to classify community pairs based on the potential influence of deterministic and stochastic processes. Changes in phylogenetic or taxonomic diversity were measured using the null-model-based phylogenetic beta diversity indicator (βNTI). The bmntd index was calculated 1,000 times using the *comdistnt* function in the *phangorn* package, followed by the calculation of βNTI values. The Raup–Crick metric based on Bray–Curtis (RCbray) was calculated using *R*.

### 2.5 Statistical analysis and machine learning model

We used analysis of variance (ANOVA) and *t*-tests to evaluate the statistical differences among groups in R. The Random Forest model was implemented using the *RandomForestClassifier* module from the *scikit-learn* (v1.2.2) package in Python with 10-fold crossvalidation to split training and test datasets. The model was configured with the following hyperparameters: number of decision trees in the forest (*n*_estimators) = 8, minimum number of samples required to split an internal node (min_samples_split) = 2, and minimum number of samples required to be at a leaf node (min_samples_leaf) = 4.

## 3 Results

### 3.1 Alpha and beta diversity of eukaryotic microbial communities across elevation gradients and soil conditions

**Figure 1:**
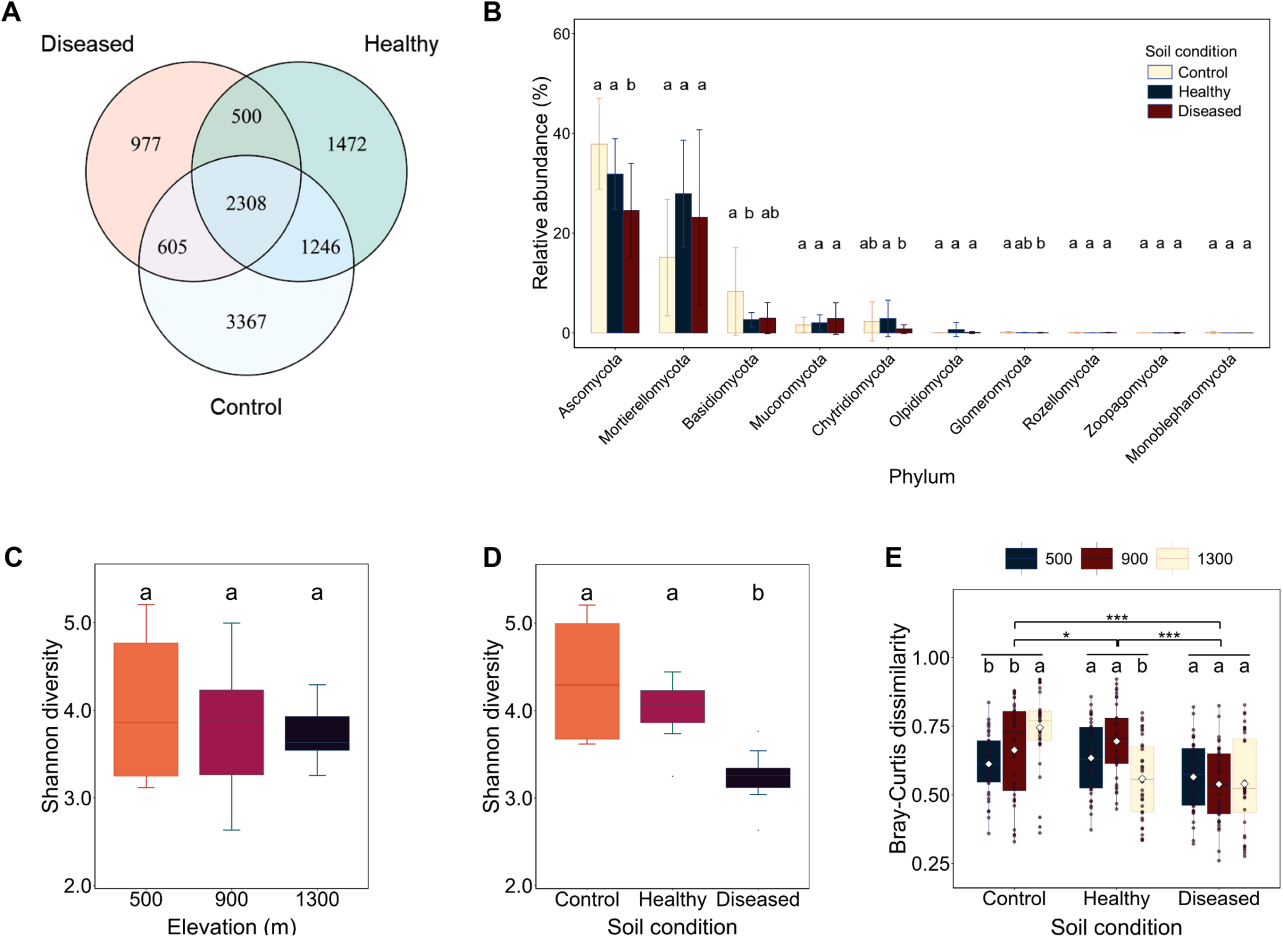
Alpha and beta diversity of eukaryotic microbial communities. **(A)** Number of shared and unique operational taxonomic units (OTUs) across three soil conditions. **(B)** The 10 major phyla of the eukaryotic microbial communities across different soil conditions. **(C)** Shannon diversity comparison across different elevations. **(D)** Shannon diversity comparison across different soil conditions. **(E)** Bray–Curtis dissimilarity comparison across different elevations and soil conditions. Conditions labeled with the same letters indicate no significant difference at *p* = 0.05, whereas conditions labeled with different letters indicate a significant difference at *p* = 0.05.

To investigate the effects of elevation gradients and soil conditions on eukaryotic microbial community diversity, we sampled three elevations (500, 900, and 1,300 m) in *Nicotiana tabacum*-cultivated areas of the Xiaogan region, Hubei Province, China. Within each elevation, rhizosphere soils were collected from three distinct soil conditions: healthy *Nicotiana tabacum* (termed “healthy”), no *Nicotiana tabacum* (termed “control”), and diseased *Nicotiana tabacum* (termed “diseased”) The ITS rDNA of eukaryotic microorganisms was amplified, sequenced, and filtered to generate 10,475 high-quality OTUs, which contained roughly 17 defined eukaryotic microbial phyla (**Supplementary Table S2**). Of these OTUs, 2,308 were shared among the three soil conditions, with the control soil harboring the highest number of unique species (3,367 OTUs), followed by healthy soil (1,472 OTUs) and diseased soil (977 OTUs) (**Figure 1A**). In all three soil conditions, *Ascomycota*, *Mortierellomycota*, and *Basidiomycota* were the most dominant phyla (**Figure 1B**).

Alpha diversity analysis was performed to compare the diversity of eukaryotic microbial communities across all OTUs. As indicated by the Shannon diversity index, the alpha diversities of microbial communities in healthy (5.21 ± 0.32) and control soils (5.18 ± 0.29) were significantly higher than in diseased soil (3.87 ± 0.41) (*p* < 0.001), suggesting that microbial diversity in diseased soil was suppressed or altered (**Figure 1D**). In contrast, the Shannon diversity index did not differ significantly across elevations (**Figure 1C**). At the phylum level, Ascomycota dominated all soil conditions (healthy: 48.2%, control: 45.7%, diseased: 62.3%), while Mortierellomycota showed a marked decline in diseased soil (healthy: 22.1% vs. diseased: 8.4%, *p* = 0.002). These results indicate that soil condition has a greater impact on the alpha diversity of eukaryotic microbial communities.

We also used Bray–Curtis dissimilarity analysis to assess beta diversity and examine the effects of elevation and soil condition on eukaryotic microbial communities. The beta diversity of the eukaryotic microbial community in diseased soil differed significantly from that in healthy and control soils. However, within the diseased soil, Bray–Curtis heterogeneity showed no significant variation across elevation gradients (**Figure 1E**). Therefore, further investigation focused solely on soil condition parameters.

### 3.2 Eukaryotic microbial community co-occurrence patterns

To analyze the topological characteristics of eukaryotic microbial communities under different soil conditions, OTUs with relative abundances greater than 0.02% in each soil condition were selected to construct co-occurrence networks and calculate various network- and node-level topological features, including average degree, graph density, and average path length.

**Figure 2:**
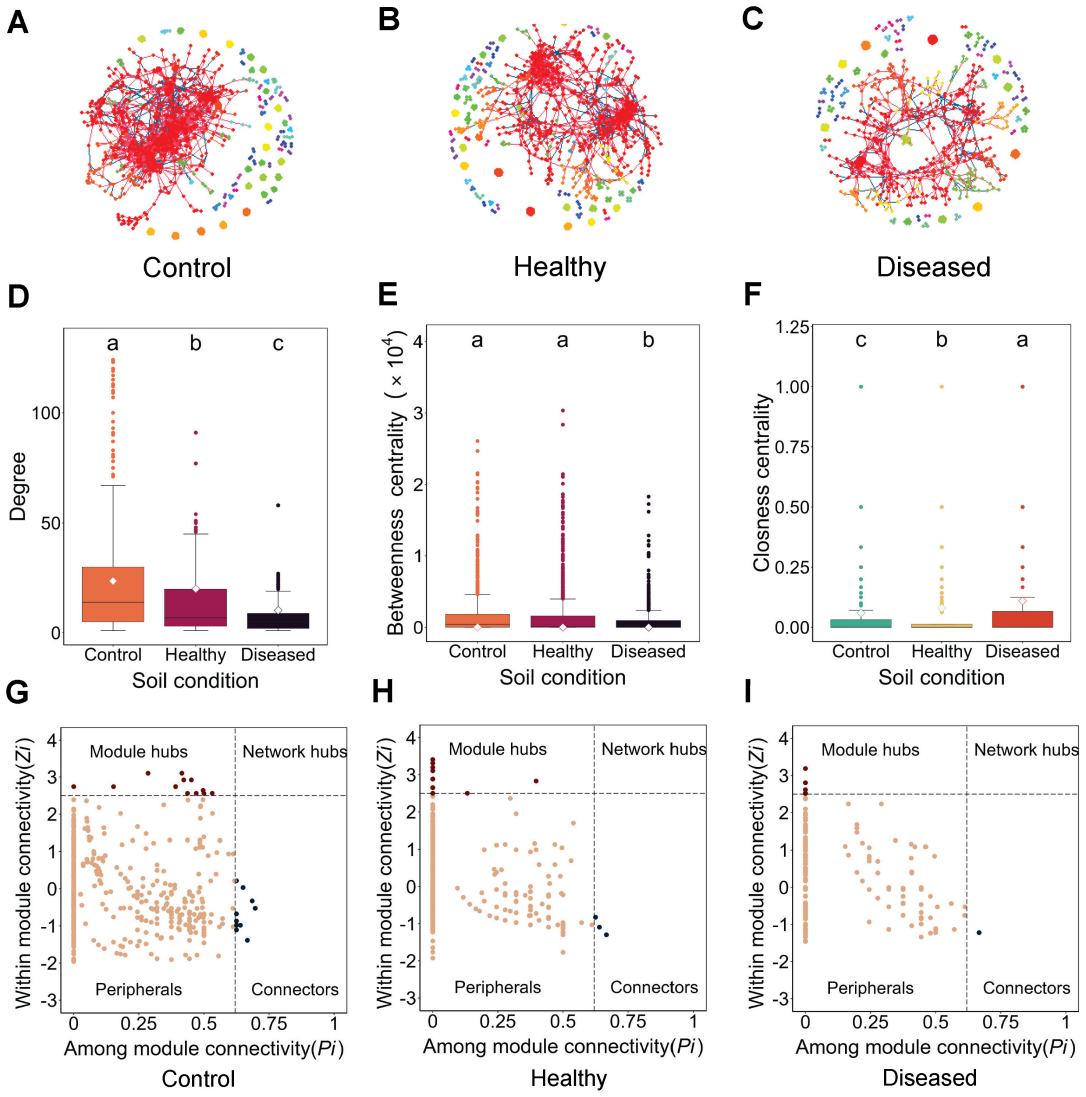
Co-occurrence network analysis. **(A–C)** Co-occurrence networks across different soil conditions: **(A)** control, **(B)** healthy, and **(C)** diseased. Red lines connecting nodes represent positive OTU connections, while blue lines represent negative connections. **(D–F)** Node-level topological feature parameters: **(D)** degree of co-occurrence network, **(E)** betweenness centrality of the co-occurrence network, and **(F)** closeness centrality of the co-occurrence network. **(G–I)** Key species analysis across different soil conditions: **(G)** control, **(H)** healthy, and **(I)** diseased. Conditions labeled with the same letters indicate no significant difference at *p* = 0.05, whereas conditions labeled with different letters indicate a significant difference at *p* = 0.05.

In all networks across different soil conditions, the OTUs, represented as nodes, were mostly positively connected (**Figures 2A–C**), while the node-level topological features varied significantly across soil conditions (**Figures 2D–F**; **Supplementary Table S3**). The eukaryotic microbial community network in diseased soil was the simplest and loosest, characterized by the lowest average degree, lowest graph density, and longest closeness centrality. In contrast, the network in control soil was the most complex and tightly connected, with the highest average degree, highest graph density, and shortest closeness centrality. The healthy soil network exhibited intermediate characteristics (**Figures 2A–F**; **Supplementary Table S3**). Using “within-module connectivity” (Zi) and “among-module connectivity” (Pi) to determine the roles of individual OTUs (represented as nodes), we found that diseased soil contained only six module hubs and one connector. In contrast, control soil had 13 module hubs and 12 connectors, while healthy soil had 17 module hubs and three connectors (**Figures 2G–I**). The reduced and dispersed interactions in diseased soil resulted in a simpler community structure, suggesting potential loss, instability, and a lack of critical hub nodes essential for maintaining community stability and function. Collectively, the structural differences in eukaryotic microbial community networks between diseased and healthy soils are evident not only in the overall community complexity but also in the interaction patterns among microorganisms.

### 3.3 Assembly processes of eukaryotic microbial communities across soil conditions

**Figure 3:**
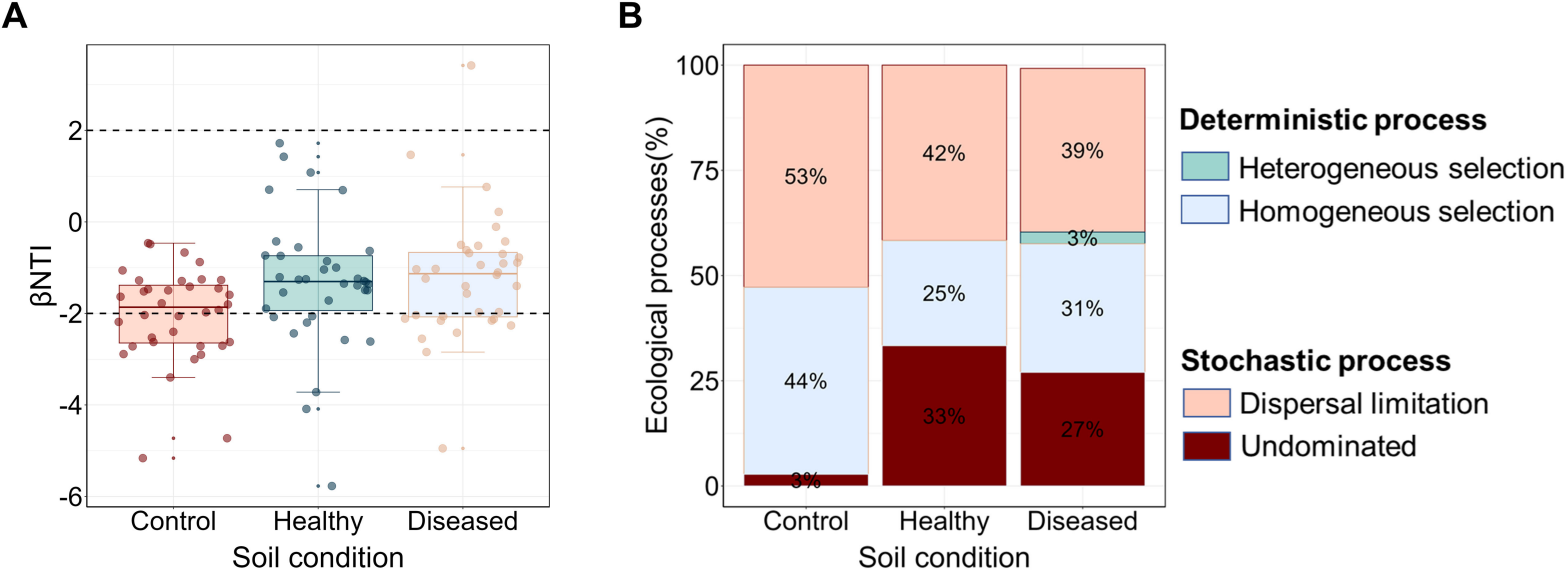
Assembly process of eukaryotic microbial community across different soil conditions. **(A)** Variations in βNTI across different soil conditions. **(B)** Contributions of various ecological processes to the assembly of eukaryotic microbial communities.

To investigate the assembly processes of eukaryotic microbial communities under different soil conditions, we employed null-model analysis and the mean nearest taxon index (βNTI) to assess the relative contributions of stochastic and deterministic processes. The results showed that in both healthy and diseased soils, most βNTI values ranged from − 2 to 2, indicating that stochastic processes predominantly drive microbial community assembly (**Figure 3A**). To further examine the role of stochastic processes in community assembly, we assessed the relative importance of each process using the Raup–Crick index (RCbary value) (**Figure 3B**). The RCbary analysis indicated that dispersal limitation was the dominant factor shaping microbial community assembly, accounting for a significant proportion in control soil (53%), healthy soil (42%), and diseased soil (39%). In contrast, undominated processes contributed only 3%, 33%, and 27% in the respective soils (**Figure 3B**). Moreover, homogeneous selection contributed to microbial community assembly in control soil (44%), healthy soil (25%), and diseased soil (31%) (**Figure 3B**).

### 3.4 Correlation analysis between eukaryotic microbial communities and different environmental factors

To identify key environmental factors influencing *Nicotiana tabacum* health status, we analyzed correlations between the eukaryotic microbiome and multiple environmental variables, including AN, AP, AK, pH, OM, TN, Ca, Mg, Fe, Mn, Cu, and Zn (**Supplementary Table S4**).

**Figure 4:**
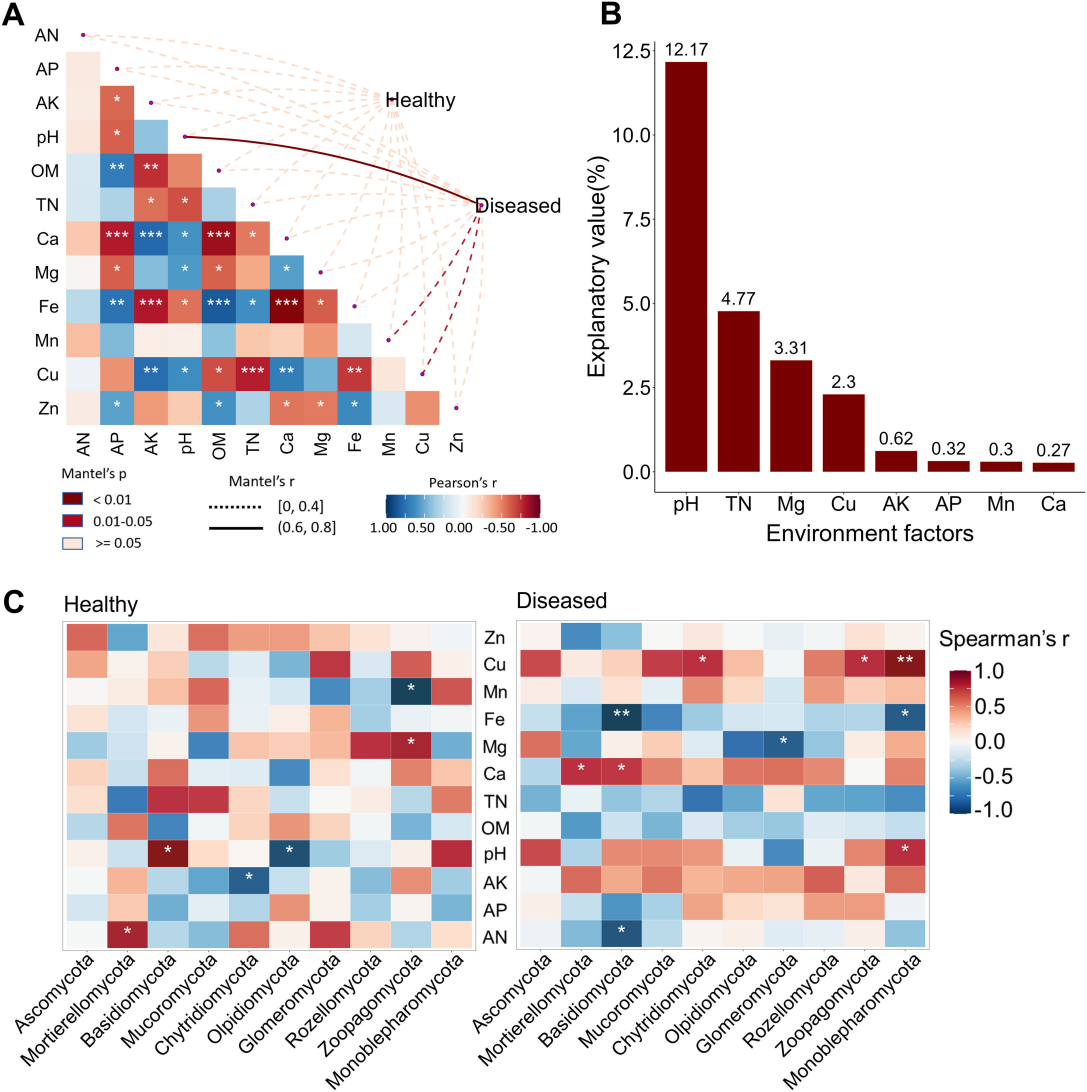
Environmental factors influencing eukaryotic microbial communities. **(A)** Environmental drivers of eukaryotic microbial communities assessed using Mantel tests in healthy and diseased soils. **(B)** Variance partitioning analysis (VPA) showing the effects of environmental factors on community structure. **(C)** Correlation between the key contributing phyla under different soil conditions and environment factors: healthy (left), diseased (right). ^***^ *p* < 0.001; ^**^ *p* < 0.01; ^*^ *p* < 0.05.

Correlation analysis revealed that pH fluctuation (*r* > 0.6, *p* < 0.01) and changes in Mn and Cu contents (0 < *r* < 0.4, *p* < 0.05) were strongly associated with the onset of *Nicotiana tabacum* disease (**Figure 4A**). To further explore the relationship between environmental factors and eukaryotic microbial communities under different soil conditions, we examined the correlations between the top 10 microbial phyla and environmental factors. In diseased soil, pH fluctuation was positively correlated with the abundance of the Monoblepharomycota phylum, whereas in healthy soil, it was positively correlated with Basidiomycota abundance and negatively correlated with Olpidiomycota abundance. Additionally, Cu content showed a positive correlation with the abundance of Chytridiomycota, Zoopagomycota, and Monoblepharomycota in diseased soil, but no such correlation was observed in healthy soil (**Figure 4C**). Furthermore, variance partitioning analysis (VPA) quantified the contribution of each environmental factor to microbial community composition, revealing that pH, TN, Mg, and Cu had the greatest impact on community structure (**Figure 4B**).

### 3.5 Machine learning model for predicting *Nicotiana tabacum* health status

**Figure 5:**
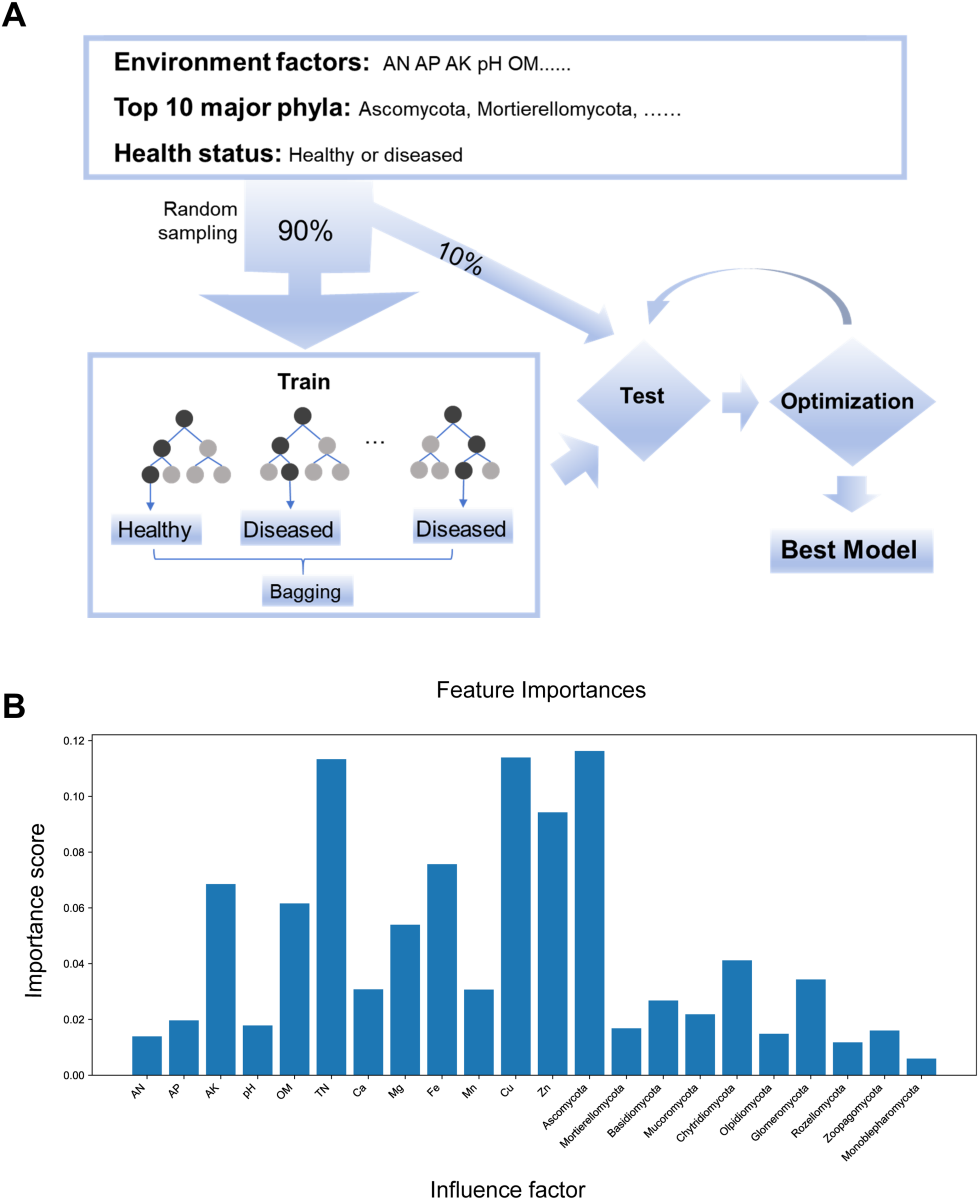
Machine learning model for predicting *Nicotiana tabacum* health status. **(A)** Schematic diagram of the machine learning model workflow. **(B)** Importance scores of influencing factors for predicting *Nicotiana tabacum* health status with random forest model.

To predict *Nicotiana tabacum* health status, a machine learning model was built using soil environment factors (AN, AP, AK, pH, OM, TN, Ca, Mg, Fe, Mn, Cu, and Zn), the top 10 major phyla (Ascomycota, Mortierellomycota, Basidiomycota, Mucoromycota, Chytridiomycota, Olpidiomycota, Glomeromycota, Rozellomycota, Zoopagomycota, and Monoblepharomycota), and binary *Nicotiana tabacum* phenotype data (healthy or diseased). Using the Random Forest (RF) algorithm with 10-fold crossvalidation, we trained and tested an optimized model, achieving 93% prediction accuracy (**Figure 5A**). Feature importance analysis using the RF model revealed that the environment factors Cu and TN, along with the major phyla Ascomycota and Chytridiomycota, are key predictors of *Nicotiana tabacum* health status (**Figure 5B**). These findings underscore the model’s high prediction accuracy and highlight the significant role of Cu, TN, Ascomycota, and Chytridiomycota in *Nicotiana tabacum* health. Monitoring and adjusting these factors could help reduce disease risks, enhance crop yield and quality, and support sustainable *Nicotiana tabacum* cultivation.

## 4 Discussion

Predicting plant health is crucial for enhancing yield and quality, particularly in economic crops (Cook, 2000). In this study, we systematically examined the relationship among *Nicotiana tabacum* health, the microbial community, and environmental factors using multiple statistical analyses, yielding a few unexpected findings.

Given that *Nicotiana tabacum* was grown at diverse elevations and previous studies have emphasized the role of elevation in microbial community diversity (Tang et al., 2020; Duan et al., 2021; Li et al., 2022; Liu et al., 2022), we aimed to determine the correlation between elevation gradients and rhizosphere microbial community composition. However, our findings indicate that elevation has a less significant influence on microbial community compositions compared to the health status of the associated *Nicotiana tabacum*. This insignificance may be due to the limited microbial diversity in the selected *Nicotiana tabacum* growing area and the relatively small elevation differences. Furthermore, in identifying key environmental factors influencing *Nicotiana tabacum* health and eukaryotic microbial community structure, we found that pH, manganese, and copper play crucial roles in disease onset, while pH and copper also impact eukaryotic microbial communities under different soil conditions. These findings are consistent with previous research indicating that soil pH and micronutrient availability significantly influence *Nicotiana tabacum* health by shaping microbial community structure (Jiang et al., 2024). Similarly, a study on tobacco black shank disease highlighted the crucial role of soil copper levels in disease suppression (Chen J-n. et al., 2022).

The decline in alpha and beta diversity of the eukaryotic microbial community in diseased soil observed in our study aligns with findings from other crops. For instance, a similar reduction in microbial diversity was reported in the rhizosphere of *Fusarium*-infected banana plants, underscoring the roles of microbial community stability in plant health (Zhou et al., 2019). Likewise, our observation of a loose microbial network structure in diseased soil parallels findings of disrupted microbial networks in the rhizosphere of wilt-infected cotton plants (Tie et al., 2023). These similarities suggest that the stability of microbial networks may serve as a general biomarker for plant health across diverse crops.

The machine learning model developed in our study integrated environmental factors and major microbial phyla information, achieving high prediction accuracy of *Nicotiana tabacum* health status. In contrast, traditional prediction models relying on single-modality data (e.g., spectral or environmental factors alone) exhibited lower accuracy. For example, Zhang et al. achieved 85% accuracy in predicting tobacco disease using only spectral data (Zhang et al., 2019). While Convolutional Neural Networks (CNNs) have proven useful for image-based plant disease diagnosis (Mohanty et al., 2016), their requirement for large labeled datasets and computational complexity limits their widespread application. Hybrid frameworks that combine our feature-driven approach with deep learning could potentially enhance prediction accuracy even further.

This study establishes the correlation between environmental factors and the health status of *Nicotiana tabacum*, as well as between plant health and the composition of the eukaryotic microbial community. Using this information and the machine learning model, future plant health can be predicted with high accuracy, providing a valuable tool for monitoring. However, further experiments are needed to establish causality between *Nicotiana tabacum* health and microbial community composition. Future work should focus on detecting and isolating health-signature taxa, with the potential to utilize them as bioprotective agents against *Nicotiana tabacum* pathogens.

## Data Availability

The data presented in the study are deposited in the NCBI repository, accession number PRJNA12277782.
