# Supplementary material for: Insights into the association of Nicotiana tabacum health with eukaryotic microbial community and environmental factors
Source: Front Plant Sci. 2025 Apr 3;16:1563283. doi: 10.3389/fpls.2025.1563283 (PMC12003371; doi:10.3389/fpls.2025.1563283)
Supplement: Supplementary file 1 [file Table1.docx]

**Supplementary table S1.** **Measured values of environment factors in the different soil conditions.**

| **AN (mg/kg)** | **AP (mg/kg)** | **AK (mg/kg)** | **pH** | **OM (%)** | **TN (%)** | **Ca (mg/kg)** | **Mg (mg/kg)** | **Fe (mg/kg)** | **Mn (mg/kg)** | **Cu (mg/kg)** | **Zn (mg/kg)** | **Soil conditions** |
| --- | --- | --- | --- | --- | --- | --- | --- | --- | --- | --- | --- | --- |
| 148.12 | 80.89 | 700.58 | 6.31 | 2.61 | 0.15 | 1617.96 | 94.02 | 40.36 | 40.81 | 1.67 | 1.47 | Healthy |
| 143.30 | 71.28 | 684.20 | 6.19 | 2.54 | 0.15 | 1566.29 | 89.68 | 40.05 | 42.37 | 1.58 | 1.65 | Healthy |
| 145.83 | 64.58 | 653.58 | 6.46 | 2.53 | 0.15 | 2021.92 | 93.60 | 41.66 | 34.87 | 1.69 | 1.93 | Healthy |
| 148.85 | 76.31 | 732.22 | 5.96 | 2.78 | 0.14 | 1739.24 | 83.85 | 44.16 | 37.20 | 1.77 | 1.62 | Healthy |
| 144.31 | 107.97 | 416.67 | 5.79 | 3.57 | 0.16 | 679.50 | 79.23 | 83.36 | 43.19 | 1.43 | 1.85 | Healthy |
| 155.40 | 114.60 | 536.03 | 5.42 | 3.80 | 0.15 | 572.79 | 75.60 | 82.60 | 43.17 | 1.59 | 1.99 | Healthy |
| 136.08 | 117.71 | 711.69 | 5.47 | 2.63 | 0.14 | 1440.96 | 88.01 | 44.73 | 38.13 | 1.64 | 1.92 | Healthy |
| 146.24 | 87.97 | 687.56 | 5.42 | 2.99 | 0.15 | 1476.41 | 94.84 | 46.68 | 34.81 | 1.67 | 1.72 | Healthy |
| 153.22 | 76.40 | 655.84 | 5.59 | 3.09 | 0.13 | 1400.10 | 90.70 | 39.33 | 39.15 | 1.54 | 1.63 | Healthy |
| 144.11 | 94.92 | 439.71 | 5.70 | 3.80 | 0.17 | 711.67 | 82.68 | 82.84 | 41.66 | 1.08 | 2.18 | Diseased |
| 143.63 | 113.34 | 307.48 | 5.79 | 3.72 | 0.18 | 640.91 | 91.70 | 79.43 | 36.66 | 1.15 | 1.93 | Diseased |
| 152.22 | 100.02 | 698.53 | 5.91 | 3.37 | 0.16 | 754.28 | 87.75 | 79.97 | 43.04 | 1.50 | 2.14 | Diseased |
| 139.78 | 69.83 | 754.86 | 5.65 | 2.97 | 0.18 | 1746.51 | 84.61 | 39.88 | 33.40 | 1.40 | 1.89 | Diseased |
| 145.47 | 126.12 | 519.4 | 5.57 | 3.44 | 0.17 | 734.54 | 74.24 | 81.81 | 41.46 | 1.45 | 2.18 | Diseased |
| 143.99 | 73.51 | 602.67 | 6.07 | 2.83 | 0.14 | 1674.77 | 81.08 | 45.84 | 36.31 | 1.54 | 1.92 | Diseased |
| 150.15 | 95.21 | 482.02 | 4.9 | 3.05 | 0.22 | 665.06 | 84.46 | 84.55 | 29.07 | 1.12 | 1.73 | Diseased |
| 153.43 | 107.72 | 427.76 | 4.84 | 3.34 | 0.24 | 709.08 | 73.72 | 86.42 | 29.69 | 1.07 | 2.04 | Diseased |
| 138.78 | 127.84 | 519.34 | 5.02 | 3.76 | 0.17 | 807.95 | 69.05 | 75.07 | 68.5 | 1.13 | 1.96 | Diseased |

**Supplementary table S2. Fungal Phyla in Diversity Analyses.**

| **Phyla** |
| --- |
| Aphelidiomycota |
| Ascomycota |
| Basidiomycota |
| Blastocladiomycota |
| Calcarisporiellomycota |
| Chytridiomycota |
| Entorrhizomycota |
| Glomeromycota |
| GS01 |
| Kickxellomycota |
| Monoblepharomycota |
| Mortierellomycota |
| Mucoromycota |
| Neocallimastigomycota |
| Olpidiomycota |
| Rozellomycota |
| Zoopagomycota |

**Supplementary table S3. The topological characteristics of eukaryotic microbial networks in different soil conditions.**

| **Topological Parameters** | **Control** | **Healthy** | **Diseased** |
| --- | --- | --- | --- |
| Average Degree | 23.66 | 20.07 | 10.35 |
| Number of Nodes | 1525 | 1236 | 787 |
| Number of Edges | 18044 | 12404 | 4072 |
| Average Path Length | 5.41 | 7.07 | 7.35 |
| Clustering Coefficient | 0.758 | 0.941 | 0.959 |
| Modularity | 0.734 | 0.777 | 0.776 |
| Positive Correlation | 0.98 | 0.97 | 0.95 |
| Negative Correlation | 0.02 | 0.03 | 0.05 |
| Centralization Degree | 0.066 | 0.057 | 0.060 |
| Centralization betweenness | 0.021 | 0.034 | 0.056 |
| Centralization Closeness | 1.10 | 1.12 | 1.07 |
| Centralization Eigenvector | 0.92 | 0.93 | 0.93 |

**Supplementary table S4. Environmental information in different soil conditions.**

| **Environment factors** | **Soil condition** | |
| --- | --- | --- |
|  | Healthy | Diseased |
| AN (mg/kg) | ^a^146.82 ± 5.65 | ^a^145.73 ± 5.18 |
| AP (mg/kg) | ^a^88.63 ± 19.80 | ^b^100.95 ± 20.46 |
| AK (mg/kg) | ^a^642.04 ± 101.62 | ^b^527.97 ± 139.13 |
| pH | ^a^5.85 ± 0.40 | ^b^5.49 ± 0.46 |
| OM (%) | ^a^2.95 ± 0.46 | ^b^3.36 ± 0.36 |
| TN (%) | ^a^0.15 ± 0.01 | ^b^0.18 ± 0.03 |
| Ca (mg/kg) | ^a^1390.57 ± 472.64 | ^b^938.31 ± 440.88 |
| Mg (mg/kg) | ^a^87.73 ± 6.82 | ^b^81.03 ± 7.33 |
| Fe (mg/kg) | ^a^51.44 ± 18.05 | ^b^72.87 ± 17.38 |
| Mn (mg/kg) | ^a^39.30 ± 3.31 | ^a^39.98 ± 11.83 |
| Cu (mg/kg) | ^a^1.62 ± 0.10 | ^b^1.27 ± 0.20 |
| Zn (mg/kg) | ^a^1.75 ± 0.18 | ^b^2.00 ± 0.15 |
